# Superpixel-Based Feature for Aerial Image Scene Recognition

**DOI:** 10.3390/s18010156

**Published:** 2018-01-08

**Authors:** Hongguang Li, Yang Shi, Baochang Zhang, Yufeng Wang

**Affiliations:** 1 Institute of Unmanned Systems, Beihang University, Beijing 100191, China; lihongguang@buaa.edu.cn; 2 Key Laboratory of Advanced Technology of Intelligent Unmanned Flight System of Ministry of Industry and Information Technology, Beijing 100191, China; 3 School of Electronic and Information Engineering, Beihang University, Beijing 100191, China; wyfeng@buaa.edu.cn; 4 School of Automation Science and Electrical Engineering, Beihang University, Beijing 100191, China; bczhang@buaa.edu.cn

**Keywords:** superpixel-based feature, image scene recognition, aerial remote sensing

## Abstract

Image scene recognition is a core technology for many aerial remote sensing applications. Different landforms are inputted as different scenes in aerial imaging, and all landform information is regarded as valuable for aerial image scene recognition. However, the conventional features of the Bag-of-Words model are designed using local points or other related information and thus are unable to fully describe landform areas. This limitation cannot be ignored when the aim is to ensure accurate aerial scene recognition. A novel superpixel-based feature is proposed in this study to characterize aerial image scenes. Then, based on the proposed feature, a scene recognition method of the Bag-of-Words model for aerial imaging is designed. The proposed superpixel-based feature that utilizes landform information establishes top-task superpixel extraction of landforms to bottom-task expression of feature vectors. This characterization technique comprises the following steps: simple linear iterative clustering based superpixel segmentation, adaptive filter bank construction, Lie group-based feature quantification, and visual saliency model-based feature weighting. Experiments of image scene recognition are carried out using real image data captured by an unmanned aerial vehicle (UAV). The recognition accuracy of the proposed superpixel-based feature is 95.1%, which is higher than those of scene recognition algorithms based on other local features.

## 1. Introduction

### 1.1. Background

Aerial remote sensing significantly complements satellite remote sensing. Recent developments in unmanned aerial vehicles (UAVs), platforms, positional and attitudinal measurement sensors, imaging sensors, and processing approaches have opened up considerable opportunities for applying remote sensing in national environmental protection [1], land use survey [2], marine environmental monitoring [3], water resource development, crop growth monitoring and assessment [4], wildlife multispecies remote sensing [5], forest protection and monitoring [6], natural disaster monitoring and evaluation [7], target surveillance [8], and Digital Earth. Subsequently, these applications have greatly promoted the development of aerial remote sensing.

Scene recognition has long been a popular and significant research field [9]. Image scene recognition, the most common task of aerial remote sensing applications, is the process of marking images according to semantic categories, such as seashore, forest, field, mountain, and city scenes. Scene recognition has also been the research focus in machine learning, computer vision, and image processing, among others.

### 1.2. Related Work

Despite the rapid development of convolutional neural networks in recent years, traditional methods for feature-based machine learning offer important application value to image scene recognition. Image feature description and extraction are core technologies for many aerial remote sensing applications. Image scene features can be described accurately by their hierarchical levels using the following techniques: scale-invariant feature transform (SIFT) [10,11] and histogram of oriented gradient (HOG) [12] for low-level features; Bag-of-Words [13,14] modeling, sparse coding [15], and deformable parts modeling [16] for middle-level features; and topic modeling [17,18] and spatial pyramid matching [19,20] for high-level features. 

Low-level features are closest to the digital storage forms of images (i.e., color, texture, and shape). Regarded the most direct source of image information, low-level features can be used to obtain higher-level information. Thus, low-level features provide the basis for image scene cognition. Middle-level features are extracted statistically or by reasonable judgment through image mining techniques. Thus, middle-level features can describe semantic content, which implies higher-level accuracy for image scene recognition. High-level features can be obtained by modeling the features of the middle layer to subsequently describe the content closest to the human perception of an image. 

Among the above feature levels and image recognition techniques, the middle-level semantic feature of the Bag-of-Words model effectively solves the “semantic gap” between low-level image information and high-level semantics. The multi-level characterization of the Bag-of-Words model is more advanced than low-level imaging techniques and is therefore widely used in scene recognition. In Bag-of-Words modeling, local features are regarded as major factors affecting scene recognition performance. Scene feature descriptions are generally divided into two kinds. The first set of descriptors is based on the detection of points of interest, such as Harris corner detector [21], features from accelerated segment test (FAST) [22], Gaussian laplacian, and Gaussian difference, among others. The other set of descriptors is based on dense extraction, such as SIFT, HOG, and extraction of local binary patterns (LBP) [23], among others. However, these methods employ bottom-to-top descriptions and consider image characteristics (point, color, texture, and shape) from a certain aspect only, thereby leading to incomplete image information. These conditions imply that even the advanced feature modeling processes (e.g., Bag-of-Words model) employ bottom-led mathematical deduction and statistics while neglecting top-led semantic expression. 

Scenes from current aerial imaging processes are often characterized as follows: (1) depth of information is lost and entire scenes are approximated as a plane given the far-off distance between the aerial equipment and captured images; (2) contents of image planes are categorized as classical types of landforms, such as grasslands, deserts, buildings, and rivers, among others; and (3) the recognized image scenes are constrained to the classical types of landforms only. Based on these delimited aerial image scene characteristics, the bottom-to-top method for low-level feature description cannot acquire local surface content. Thus, the Bag-of-Words model is not as effective when dealing with scene recognition.

### 1.3. Contribution of This Paper

This study first considers aerial image landforms for scene identification. Then, the study proposes a top-down feature-based image scene recognition method for aerial application. 

The main work and contributions of this study are as follows: (1) A novel superpixel-based feature description method is proposed. To utilize landform information, the study establishes the features acquired by top-task landform superpixel extraction to bottom-task expression of feature vectors. (2) Using the proposed superpixel-based features, a type of Bag-of-Words model is constructed and an image scene recognition algorithm is designed. (3) Experiments are carried out using real image data captured by UAVs.

## 2. Methodology

### 2.1. Aerial Image Semantic Hierarchical Structure

To better demonstrate the motivation and basis of the proposed method, this section introduces a hierarchical structure model of aerial image semantics. 

**Figure 1:**
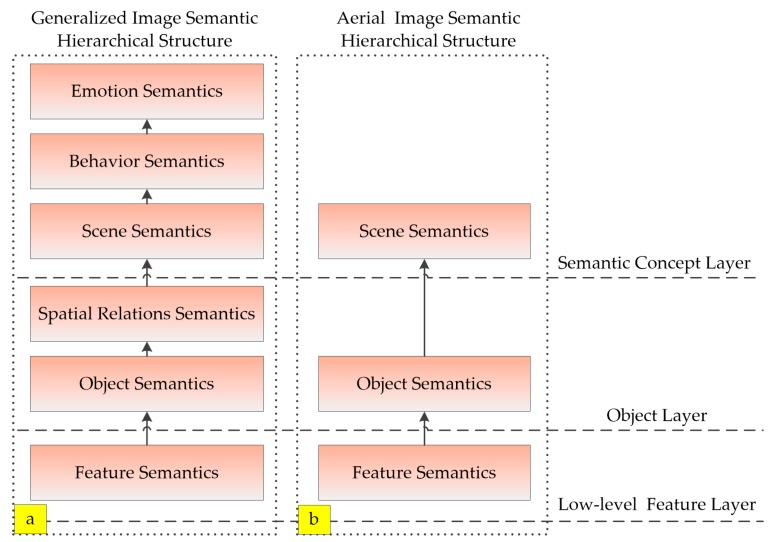
Image semantic hierarchical structure: (**a**) generalized image semantic hierarchical structure; and (**b**) aerial image semantic hierarchical structure.

Image semantics is the description of the image content itself. According to the abstraction degree of image semantics, Eakin [24] divides it into 3 layers: low-level feature layer, object layer and semantic concept layer. From bottom to top, it includes feature semantics, object semantics, spatial relations semantics, scene semantics, behavior semantics and emotion semantics, as shown in Figure 1a. High level semantics are often more abstract than low-level semantics and are quantified by a lower level of semantics. 

Because of the characteristics of long-distance imaging, there are few information about spatial relations semantics, behavior semantics and emotional semantics in aerial images. Its main semantic features are feature semantics, object semantics and scene semantics, as shown in Figure 1b. Feature semantics include texture, color, shape, structure and so on, which correspond to the basic visual perception. The object semantics is embodied in the landform area or target area of the aerial image, which can be used to model the semantic features for scene and target recognition. Scene semantics refers to the image scene label, which involves a higher level of abstract attributes, and is derived from the object semantics.

Based on the above aerial image semantic hierarchical structure, a novel superpixel-based feature is proposed in this paper, which corresponds to the object semantics in the semantic structure. In the process of proposed feature based image scene recognition, landform is a useful and basic clue for method design. In the object semantic layer, superpixel is used to express landform information in Section 2.2.1. Then, Section 2.2.2, Section 2.2.3 and Section 2.2.4 describe how to extract the low-level landform feature of superpixel region, which corresponds to the feature semantics in the semantic structure. Finally, Section 2.3 gives the overall flow of the scene recognition of aerial images.

### 2.2. Superpixel-Based Feature Description

**Figure 2:**
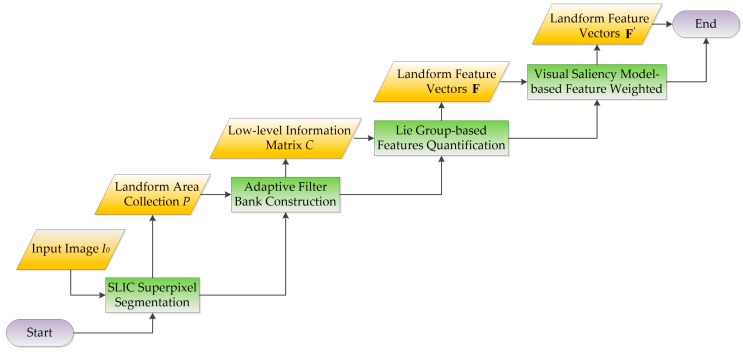
Flowchart of superpixel-based feature extraction (green: process; yellow: data type).

A novel process for superpixel-based feature description is proposed for capturing landforms aerially during scene identification. The process mainly consists of the following steps: simple linear iterative clustering (SLIC) [25] based superpixel segmentation, adaptive filter bank construction, Lie group-based feature quantification, and feature weighting based on the visual saliency model (Figure 2). SLIC is used to extract superpixels, whereas filter banking, feature quantification, and feature weighting are conducted to transform the superpixels into feature vectors.

In Figure 2, input image $I_{0}$ is initially segmented by SLIC superpixel algorithms to collect landform data of the image. Based on on prior landform information, 2D linear discriminant analysis [26,27] is conducted to adaptively construct the filter bank. Second, the filter bank is convolved with the image to obtain matrix *C* with low-level information (color, texture, context, etc.). Each pixel corresponds to a filter response vector, and the image content of the irregular surface is described by a feature matrix that comprises the corresponding feature vectors of the pixels. Third, the Lie group theory based on Riemann manifold geometry [28,29,30,31] is used to analyze the topological relations of surface pixels, and then the feature matrix is mapped into vector space to generate feature vector F. Finally, local feature vector $F^{\prime }$ is obtained by weighting the feature vectors according to the visual saliency model.

#### 2.2.1. SLIC Superpixel Segmentation

Aerial image scenes differ from low-altitude outdoor images. Scenes in low-altitude outdoor images are mostly composed of a background and a target, which are defined differently. Backgrounds may include skies, roads, grasslands, and buildings; by contrast, targets may include pedestrians and vehicles. Objects such as pedestrians and vehicles are proportionally small compared with the image because of the far-off imaging distance of aerial platforms; thus, the depths of field of image planes are nearly the same. Image scenes are generally composed of different landforms, such as grasslands, deserts, buildings, rivers, and so on. Therefore, landforms comprise the basic components of scene semantics of aerial images.

Statistical analyses of aerial images generally depict landforms as irregularly shaped images with different colors and textures. In particular, the superpixels of images comprise irregular pixel blocks with a certain visual sense, i.e., adjacent pixels with similar textures, colors, brightness and other features. Thus, the superpixel area of aerial images can represent actual landform surfaces. 

This study uses color and space distance based on the SLIC algorithm [25] to segment aerial images into landform areas. The specific steps of SLIC superpixel segmentation are as follows: 

(1) Cluster center initialization is conducted by assuming N pixels in the image. The size of each superpixel is approximately *N*/*K* after setting the number of superpixels to *K*. To avoid clustering at the edge of an object, the initial cluster is “centered” to the position wherein the gradient value is smallest, for instance, at the center of a 3×3 neighborhood window. The image gradient calculation formula is

(1) $$G\left(x,y\right)={\left\|v\left(x+1,y\right)-v\left(x-1,y\right)\right\|}^{2}+{\left\|v\left(x,y+1\right)-v\left(x,y-1\right)\right\|}^{2}$$

where v(x,y) is the pixel value at point (*x*, *y*). The cluster center is defined by ($O_{i}$, i=1,…,K).

(2) Similarity measurement in SLIC is expressed by the following:

(2) $$d\left(i,k\right)=d_{lab}+\frac{m}{S}d_{xy}$$

(3) $$d_{lab}=\sqrt{{\left(l_{k}-l_{i}\right)}^{2}+{\left(a_{k}-a_{i}\right)}^{2}+{\left(b_{k}-b_{i}\right)}^{2}}$$

(4) $$d_{xy}=\sqrt{{\left(x_{k}-x_{i}\right)}^{2}+{\left(y_{k}-y_{i}\right)}^{2}}$$

where $d_{lab}$ is the color difference between two pixels; *l*, *a*, and *b* are the three components of Lab color space; $d_{xy}$ is the spatial distance between two pixels; (*x*, *y*) is pixel position; d(i,k) is the similarity between *i*th pixel and *k*th cluster center (i.e., the smaller the value, the higher the similarity); and m∈[1,20] is the parameter that balances color and spatial information in the similarity measure, which is set to 10 in this study. The desired superpixel size is S×S, where $S=\sqrt{N/K}$.

**Figure 3:**
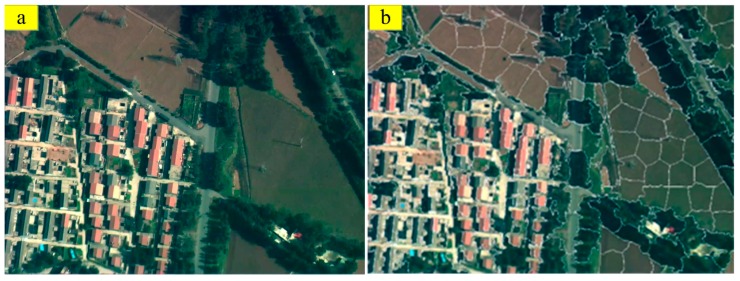
Superpixel segmentation of an aerial image: (**a**) aerial image; and (**b**) result of superpixel region segmentation of (**a**).

(3) The *K*-means clustering method involves an iteration of the clustering center. Based on the measured similarity, the *K*-means clustering method is performed on the 2S×2S region in the X – Y image plane. The process is repeated until convergence (i.e., the maximum number of times) is reached. The initial cluster centers are uniformly initialized in the image with all pixels situated near their cluster center. 

(4) The process of generating the superpixel regions is conducted given that some small areas may be present in the regions. Each generated small area is labeled as a superpixel even if these areas are not connected to the superpixel. The small areas are thus connected to the largest superpixel to ensure the integrity of every superpixel. For instance, Figure 3b is the result of the superpixel region segmentation of Figure 3a.

#### 2.2.2. Adaptive Filter Bank Construction

Different types of landforms in aerial images vary greatly in color, texture, and shape. To fully describe landform features, the aerial images are filtered and low-level feature modeling is performed using a filter bank. Filter banks are an array of band-pass filters that separate input signal from multiple components. Filter types, scale parameters, and direction parameters are determined in accordance with the actual situation on the basis of different task requirements. 

In the construction of the filter bank, any prior information about the surface is assumed to have five types of image landforms, namely, buildings, trees, grasses, bare lands, and roads. Then, 2D linear discriminant analysis [27] is conducted on the basis of the surface prior information to adaptively construct image filter banks and their weight templates. Consequently, the filter banks are convolved with the input image to generate low-level features.

If image $I_{0}$ is filtered by filter $F_{0}$, then the response image is $I_{0}^{\prime }$. Then, filtering response $r^{\left(\psi \right)}$ corresponding to point ψ in response image $I_{0}^{\prime }$ is regarded a convolution value of filter $F_{0}$ and the image area in which point ψ is contained. The process can be expressed as Equation (5).

(5) $$r^{\left(\psi \right)}=F_{0}{}^{T}p_{\psi }$$

where $F_{0}{}^{T}\in {\mathbb{R}}^{L_{n}\times 1}$ represents the vector form of filter $F_{0}$ and $p_{\psi }$ represents the vector form of the image region in which point ψ is the center.

**Figure 4:**
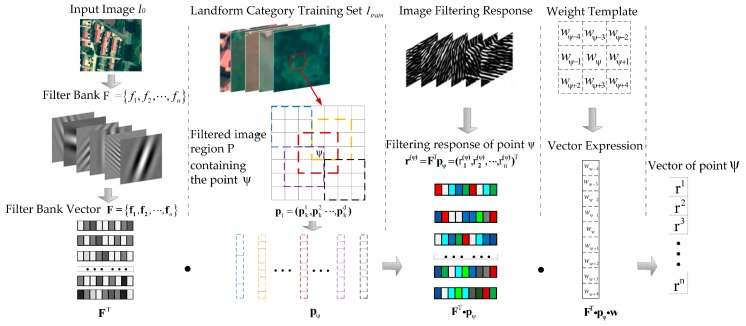
Adaptive filter bank construction. Filter banks are convolved with input image $I_{0}$, to generate low-level feature ψ.

The constructed adaptive filter bank and corresponding processes are shown in Figure 4. 

In Figure 4, $F=\left\{f_{1},f_{2},\ldots ,f_{n}\right\}$ is the vector expression of filter bank $F=\left\{f_{1},f_{2},\ldots ,f_{n}\right\}$. After the convolution of $F=\left\{f_{1},f_{2},\ldots ,f_{F}\right\}$ and image $I_{0}$, the corresponding filtering response of point ψ is defined as Equation (6).

(6) $$r^{\left(\psi \right)}=F^{T}p_{\psi }={\left(r_{1}^{\left(\psi \right)},r_{2}^{\left(\psi \right)},\ldots ,r_{n}^{\left(\psi \right)}\right)}^{T}\;\forall 1\leq k\leq n\;r_{k}^{\left(\psi \right)}=f_{k}{}^{T}p_{\psi }$$

After weighted calculation, the corresponding feature vectors of point ψ are obtained by Equation (7).

(7) $$r_{\psi }=\frac{1}{d}\sum_{i\in {\mathbb{R}}^{\left(\psi \right)}}{\left(r_{1}^{\left(\psi \right)},r_{2}^{\left(\psi \right)},\ldots ,r_{n}^{\left(\psi \right)}\right)}^{T}=\frac{1}{d}\sum_{i\in {\mathbb{R}}^{\left(\psi \right)}}F^{T}p_{i}$$

where *d* is filter template size, while $p_{i}=(p_{k}^{1},p_{k}^{2}\ldots ,p_{k}^{d})$ is the vector form of the corresponding region of an image containing point ψ. The pixels in different positions affect the feature vectors of ψ points differently. Subsequently, weight template w is defined for the obtained feature vectors, and the corresponding vector form is $w=(w_{1},w_{2},\ldots ,w_{d})$. The feature vector value of point ψ is expressed as Equation (8).

(8) $$r_{\psi }=F^{T}p_{i}w$$

To ensure that the extracted feature vectors contain abundant image information and can strongly express the surface, all prior information about the aerial landform image is used for low-level feature extraction. Based on the Fisher criterion [32], the 2D-LDA method is used to adaptively learn the filter bank and extract the low-level features of the landforms.

Variable $r_{m}^{n}$ is defined as the vector form of the *n*th image region extracted from the *m*th landform category. Intra-class and inter-class differences are quantified by Equation (9).

(9) $$\left\{ \begin{array}{l} L_{\mathrm{intra}}=\sum_{m=1}^{M}\sum_{n=1}^{N}\left(r_{m}^{n}-\bar{r_{m}}\right){\left(r_{m}^{n}-\bar{r_{m}}\right)}^{T}\\ L_{\mathrm{inter}}=\sum_{m=1}^{M}N_{m}(\bar{r_{m}}-\bar{r}){\left(\bar{r_{m}}-\bar{r}\right)}^{T} \end{array}\right.$$

where M represents surface type; $N_{m}$ represents the number of features extracted from the *m*th landform category; $\bar{r_{m}}$ is the mean of all feature vectors in the *m*th landform category; and $\bar{r}$ represents the mean of all feature vectors. After the derived feature vector formula is added to the model, intra-class and inter-class differences are determined as Equation (10).

(10) $$\left\{ \begin{array}{l} L_{\mathrm{intra}}=\sum_{m=1}^{M}\sum_{n=1}^{N}F^{T}(P_{m}^{n}-\bar{P_{m}})ww^{T}{\left(P_{m}^{n}-\bar{P_{m}}\right)}^{T}F\\ L_{\mathrm{inter}}=\sum_{m=1}^{M}N_{m}F^{T}(\bar{P_{m}}-\bar{P})ww^{T}{\left(\bar{P_{m}}-\bar{P}\right)}^{T}F \end{array}\right.$$

where $P_{m}^{n}$ represents the vector form of the corresponding region of the *n*th image in the *m*th landform category training set $I_{train}$; $\bar{P_{m}}$ represents the mean value of the *m*th category; and $\bar{P}$ is the mean value of the entire dataset.

Then, filter bank F and its weighting template w are solved by the objective functions of intra-class difference and inter-class difference, as shown in Equation (11). 

(11) $$J(F,w)=\underset{\left(F,w\right)}{\arg\max}\frac{\sum_{m=1}^{M}N_{m}F^{T}(\bar{P_{m}}-\bar{P})ww^{T}{\left(\bar{P_{m}}-\bar{P}\right)}^{T}F}{\sum_{m=1}^{M}\sum_{n=1}^{N}F^{T}(P_{m}^{n}-\bar{P_{m}})ww^{T}{\left(P_{m}^{n}-\bar{P_{m}}\right)}^{T}F}$$

Equation (11) can be solved by the 2D-LDA algorithm. Equations (12) and (13) are used as optimization functions to solve F and w. 

(12) $$\{\begin{array}{c} J(F)=\max\mathrm{trace}(F^{T}L_{\mathrm{intra}}^{w}F{)}^{-1}(FL_{\mathrm{inter}}^{w}F^{T})\\ L_{\mathrm{intra}}^{w}=\sum_{m=1}^{M}\sum_{n=1}^{N}\left(P_{m}^{n}-\bar{P_{m}}\right)ww^{T}{\left(P_{m}^{n}-\bar{P_{m}}\right)}^{T}\\ L_{\mathrm{inter}}^{w}=\sum_{m=1}^{M}N_{m}(\bar{P_{m}}-\bar{P})ww^{T}{\left(\bar{P_{m}}-\bar{P}\right)}^{T} \end{array}$$

(13) $$\{\begin{array}{c} J(w)=\max\mathrm{trace}(w^{T}L_{\mathrm{intra}}^{F}w{)}^{-1}(wL_{\mathrm{inter}}^{F}w^{T})\\ L_{\mathrm{intra}}^{F}=\sum_{m=1}^{M}\sum_{n=1}^{N}{\left(P_{m}^{n}-\bar{P_{m}}\right)}^{T}FF^{T}(P_{m}^{n}-\bar{P_{m}})\\ L_{\mathrm{inter}}^{F}=\sum_{m=1}^{M}N_{m}{\left(\bar{P_{m}}-\bar{P}\right)}^{T}FF^{T}(\bar{P_{m}}-\bar{P}) \end{array}$$

The final filter bank F and its weight template w can be obtained after multiple iterations of the optimization function. Then, filter bank F and weight template w are convolved with image $I_{0}$ to obtain the filter response image set $I_{\mathrm{res}}=\left\{I_{1},I_{2},\ldots ,I_{F}\right\}$. The filter bank is usually convolved with a gray image of the input image for low-level feature extraction. However, low-level features can only describe the texture, context, and other information of an image, but cannot reflect color characteristics. To solve this problem, this study combines nine channel images of the input image corresponding to RGB, HSV, and Lab color space with the filter response image to form the image feature set $I_{\mathrm{fea}}=\left\{I_{1},I_{2},\ldots ,I_{F},I_{F+1},\ldots ,I_{F+9}\right\}$.

#### 2.2.3. Lie Group-Based Feature Quantification

The aerial images are divided into a number of superpixel regions in which every pixel point corresponds to a multidimensional feature vector. Inputs to the Bag-of-Words model usually take the form of a feature expression vector of a local region. Thus, to effectively integrate low-level features to the Bag-of-Words model, the feature matrix describing the superpixel region is quantized as a feature vector. 

This study therefore introduces the Lie group structure [28] to the Riemann manifold space [30]. The Lie group can reflect the correlation between two feature vectors of each pixel and describe the spatial structure information of all the pixel feature vectors in the entire superpixel region. 

A total of *N* superpixels pixels in an input image can be extracted after superpixel segmentation. An arbitrary superpixel $\left\{P_{i}\in I,i=1,\ldots ,N\right\}$ in an image contains C pixels $\left\{x_{j}\in {\mathbb{R}}^{k\times 1},j=1,\ldots ,C\right\}$, and the superpixel region is expressed by the Gaussian model in accordance with the maximum likelihood method (Equation (14)).

(14) $$\phi (x_{i}|\mu ,\Sigma )=\frac{\exp(-\frac{1}{2}{\left(x_{i}-\mu \right)}^{T}\Sigma^{-1}(x_{i}-\mu ))}{\sqrt{{\left(2\pi \right)}^{k}\det(\Sigma )}}$$

where $\mu =\frac{1}{N}\sum_{i=1}^{N}x_{i}$ and $\Sigma =\frac{1}{N-1}\sum_{i=1}^{N}\left(x_{i}-\mu \right){\left(x_{i}-\mu \right)}^{T}$ are the mean value and covariance of the vector while det(⋅) is the matrix determinant. The Gaussian model with K dimensions is a Riemann manifold, and ϕ(μ,Σ) is defined as the Gaussian model with μ mean value and Σ covariance. For any random vector of the Gaussian distributions, a unique transformation can be used to meet the requirements of Equation (15).

(15) $$\left[\begin{array}{l} x\\ 1 \end{array}\right]=\left[\begin{array}{ll} H & \mu \\ 0 & 1 \end{array}\right]\left[\begin{array}{c} x_{0}\\ 1 \end{array}\right]$$

where H is an upper triangular matrix that satisfies $\Sigma =HH^{T}$. Covariance matrix Σ and H are both positive definite. Thus, the upper triangular positive definite affine matrix can be expressed as Equation (16).

(16) $$M=\left[\begin{array}{ll} H & \mu \\ 0 & 1 \end{array}\right]$$

A double shooting may occur between matrix **M** and Gaussian function. According to Lie group theory, an invertible affine scaling matrix can form a Lie group. Thus, the upper triangular positive definite affine matrix is assumed equivalent to the Gaussian function. After affine transformation, ϕ(μ,Σ) can be uniquely transformed into (k+1)×(k+1) dimensional positive definite symmetric matrix, as expressed in Equation (17).

(17) $$\phi (\mu ,\Sigma )\sim S=\left[\begin{array}{ll} \Sigma +\mu \mu^{T} & \mu \\ \mu^{T} & 1 \end{array}\right]$$

The vector space $S_{k+1}^{+}$ of positive definite symmetric matrices with (k+1)×(k+1) dimensions is a Lie group and a Riemann manifold with local topology properties equivalent to vector space. For any element in the Lie group, a corresponding Lie algebra exists in which the tangent space is a vector space. The elements of the Gaussian Lie group can be mapped between tangent space (Gaussian Lie algebras) and manifolds (Gaussian Lie group) by means of log operations and exponential matrix operations. Finally, by adopting upper triangular positive definite affine matrix transformation, the (k+1)(k+2)/2 (i.e., 190) dimensional feature vector is obtained for describing superpixel region $P_{i}$. Consequently, the final input image is expressed by *N* 190-dimensional manifold-based superpixel feature $f_{i}$.

#### 2.2.4. Visual Saliency Model-Based Feature Weighting

For two images belonging to different categories but with similar contents, the contents of the salient regions often serve an important basis for determining image categories. To enhance the expression capability of low-level features and image semantic features, a feature weighting method based on saliency is proposed in this study for aerial scene recognition. 

The steps for the algorithm are as follows: A visual saliency map $S_{map}$ with the same size as the input image is obtained according to the visual saliency model. For the feature $f_{i}$ of superpixel region $P_{i}$, the feature weight $w_{i}$ is defined as the average value of the saliency map corresponding to all pixels in the superpixel region, as shown in Equation (18).

(18) $$w_{i}=mean(S_{map}\{P_{i}\})$$

The feature corresponding to each area $P_{i}$ of the landform is $w_{i}*f_{i}$.

### 2.3. Scene Recognition of Aerial Images

The image scene recognition algorithm based on the Bag-of-Words model is determined using the proposed superpixel-based feature for aerial remote sensing applications. 

**Figure 5:**
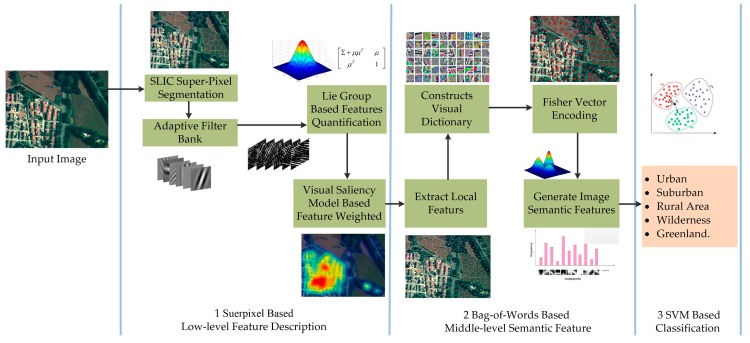
Scene recognition of aerial images. The method includes three steps: superpixel-based low-level feature extraction, Bag-of-Words model-based middle-level semantic feature extraction, and SVM-based classification.

The Bag-of-Words model constructs a visual dictionary using the low-level features of an image; performs feature coding to obtain middle-level semantic features; and integrates a classifier to realize image scene recognition. As shown in Figure 5, the three main steps of Bag-of-Words modeling are as follows: first, the superpixel-based low-level features are extracted; second, the dictionary is generated; and, finally, image scene classification is conducted.

In the first step, superpixel-based low-level features are extracted using the proposed method. In the second step, the main feature encoding methods (vector quantization, sparse, local linear constraint, Fisher vector [32], etc.) are conducted for Bag-of-Words modeling. Fisher vector encoding, which simultaneously conducts generative and discriminative modeling to record first- and second-order differences between a local feature and its nearest visual words, is generally considered for its strong capability to express features. Thus, Fisher vector encoding is selected to further quantify the local features of the extracted aerial image and generate semantic features to complete the aerial image scene recognition. In the third and final step, the SVM classifier is used to recognize five image scenes (i.e., urban, suburban, rural, wilderness, and green land). 

## 3. Result and Discussion

### 3.1. Experimental Data

**Figure 6:**
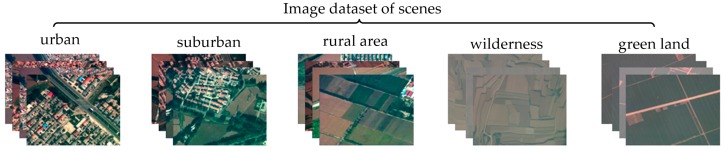
Image dataset of scenes. The designated categories are urban, suburban, rural, wilderness, and green land.

**Figure 7:**
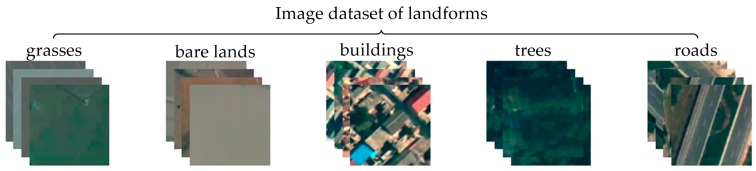
Image dataset of landforms. The landforms are categorized as buildings, trees, grasses, bare lands, and roads.

The experimental data used in this study are collected from UAV images. The dataset for scene recognition is divided into five categories, each containing 100 images (30 images for training and 70 images for testing). The designated scene categories are urban, suburban, rural, wilderness, and green land, as shown in Figure 6. The landform images used to train adaptive filters are collected from the training set of scene recognition data. Each landform image has the size of 100 × 100 (resolution), and landforms are categorized as buildings, trees, grasses, bare lands, and roads, as shown in Figure 7.

### 3.2. Experimental Results Analysis

This study applies Bag-of-Words model-based scene recognition and compares this technique with typical feature-based Bag-of-Words modeling to verify the expression capability of the proposed superpixel-based features. Model performance is evaluated in terms of scene recognition accuracy and extraction time. Filter template size and saliency model are considered during feature extraction, as both are regarded as important factors that can affect feature expression. 

#### 3.2.1. Influence of Filter Template Size on Feature Expression

The 2D linear discriminant analysis is conducted based on prior image surface information, which is also used to construct the image filter bank. Then, the filter bank is convolved with the image to obtain the low-level features of the image. This method does necessarily consider filter type, scale factor, and direction factor; however, filter template size needs to be artificially determined. This requirement suggests that filter template size can influence the capability of the proposed method to express the local features and semantic features of the image; moreover, the level of influence can be reflected by the results of the final scene recognition. The experimental results are shown in Table 1. Experiments for the low-level features are carried out under non-overlapping saliency weight conditions.

Experimental results show that filter template size has little influence on low-level and semantic features. Differences in scene recognition accuracy for the different templates are below 1%. The situation can be explained by large single superpixel areas (i.e., segmentation result), whereas filter template size is small. Image filtering is usually conducted in one-pixel steps; therefore, pixel filter response is related only to neighboring image regions and the filter's own parameters. In addition, different filter templates are trained using the same training dataset, and this approach leads to similar filtering responses despite the different size templates in the same location.

#### 3.2.2. Influence of Saliency Model on Scene Recognition

To test the influence of the saliency model on scene recognition, this study introduces four kinds of saliency models for comparison, namely, the Itti model [33], Erdem model [34], Achanta model, [35] and SIM model [36].

The Itti visual saliency model [33] is based on the three characteristics of brightness, color, and direction, and then the salient region of an image is determined after normalization. The color difference of different landforms is large, and artificial building parts (buildings and roads) have relatively high reflection coefficients than natural surfaces; thus, the brightness varies greatly for different regions in aerial images. Erdem [34] introduced region covariance into visual saliency to improve model robustness by nonlinear feature fusion. Subsequently, the capability of local features to describe image content is strengthened. The visual saliency model proposed by Achanta [35] theoretically starts with a frequency domain. Then, the saliency value of each pixel is set as the Euclidean distance between two pixel values after Gaussian low-pass filtering and pixel value averaging of the entire image in Lab space. The method uses fixed value filtering. When the salient object is relatively large, the calculation of the Lab space mean is affected; consequently, the saliency value of the salient region is less than that of the background. No obvious differences exist between target and background in aerial images, and salient areas mostly comprise artificial buildings and roads. When the proportion of the building or road to the image is extremely large, the effect of local features weighting using the salient model is unsatisfactory. To a certain extent, the category differentiation of image scenes is reduced, thereby weakening the final expressive capability of the image semantic features. SIM saliency maps [36], which are based on mathematical and statistical methods, adopt biological bottom visioning using multi-scale weight optimization for feature extraction. The method effectively captures landform information in aerial images, and the multi-scale weight optimization method can well adapt to landform regions with different scales. Therefore, the capability to express image features is enhanced to a great extent.

The influence of feature weights (i.e., extracted from different saliency maps) on the scene recognition of aerial images is shown in Table 2. Filter template size is specified as 9 × 9. According to the four typical saliency models, feature weighting can be designed to complete local feature modeling. In this study, SIM is used for the proposed algorithm.

#### 3.2.3. Comparison of Feature Performances

This study evaluates and compares the expression capability of the proposed superpixel-based features with the six commonly used features of Bag-of-Words modeling (i.e., dense SIFT, dense HOG, dense LBP, dense Gabor, Harris interest points, and FAST interest points). The final result is measured in terms of scene recognition accuracy and time consumption to extract the local features of each image, as shown in Table 3.

Dense local features were compared with interest point local features. All of the dense features, except that of dense Gabor, showed stronger expression capability than those of interest points. The condition can be explained by the sparsely distributed local features of the interest points, which resulted in limited image information. The information loss effect is further expanded after quantifying the Bag-of-Words model, the analysis of which showed a decline in semantic feature expression capability. Gabor features can comprehensively extract low-level image information using time and frequency domains. The Gabor filter sifts dense image blocks in several directions and scales in the dense modeling of local image regions; however, redundant information is generated, and this phenomenon weakens feature expression. 

The capability of dense local features to describe image content is weaker than that of the proposed feature. The phenomenon can be explained by features (i.e., dense local features) that are mainly extracted by traversing image blocks, and this approach cannot accurately capture local surface areas. This dense local feature method also often uses a single feature descriptor to model the local area of images. However, the local content of the image cannot be fully covered; thus, the capability to generate image semantic features is weaker than the method proposed in this study. 

The number of extracted dense local features is much larger than the number of interest point features because aerial image sizes are frequently large. Therefore, in terms of time consumption, the extraction of dense local features consumes much more time than those of interest point features. With regard to local feature modeling, image segmentation and saliency weighting extraction are required by the proposed method, and these additional processes may lead to relatively high time complexity. However, final results verify the high recognition accuracy of the proposed method. Moreover, the time consumption of the proposed method has not increased significantly unlike the dense SIFT, dense HOG, and dense LBP methods. The time consumption of the proposed method is even lower than that of dense Gabor features.

## 4. Conclusions

The superpixel-based feature description method proposed in this study can be applied to image scene recognition for aerial remote sensing applications. The image scene recognition algorithm based on the Bag-of-Words model is designed using the proposed feature. The following conclusions can be drawn from the experiments: (1) The proposed superpixel feature can be extracted adaptively by using the landform characteristics of aerial images. Image saliency, which is highly adaptable to aerial images, is introduced into the algorithm to complete the local area modeling. (2) The scene recognition accuracy of aerial images based on the proposed feature is 95.1%, and this result is higher than those of scene recognition algorithms based on other local features. 

In the future, the proposed method can be improved and applied to other types of remote images, such as satellite images. Finally, the method may also be transplanted to embedded systems.

## Figures and Tables

**Table 1:** Influence of filter template size on recognition results.

**Template Size**	3 × 3	5 × 5	7 × 7	9 × 9
**Recognition Accuracy**	88.3%	88%	88.3%	88.9%

**Table 2:** Influence of saliency models on recognition accuracy.

Saliency Model	Null	Itti	Erdem	Achanta	SIM
Recognition Accuracy	88.9%	92.1%	94.2%	85.4%	95.1%

**Table 3:** Comparison and analysis of local feature description methods.

Local Feature Type	Recognition Accuracy	Time Consumption
Dense SIFT	78.3%	16.86 s
Dense HOG	79.1%	15.23 s
Dense LBP	82.6%	15.96 s
Dense Gabor	72.8%	45.75 s
Harris Interest Points	73.5%	0.73 s
FAST Interest Points	78.2%	0.53 s
**Proposed Feature**	**95.1%**	21.57 s
